# Formation of the Structure and Properties of Building Ceramics Based on Coal Ash and Metallurgical Slags: A Review of Modern Research

**DOI:** 10.3390/ma19122497

**Published:** 2026-06-10

**Authors:** Madeniyet Yelubay, Tatyana Vakalova, Dias Tolegenov, Sabit Maussumbayev, Nurdana Kanasheva, Gulzat Aitkaliyeva, Sofya Massakbayeva

**Affiliations:** 1 Department of Chemistry and Chemical Technologies, Toraighyrov University, Lomov Street, 64, Pavlodar 140008, Kazakhstan; yelubay.m@tou.edu.kz (M.Y.); kanashevanur94@gmail.com (N.K.); sofyamassakbayeva@gmail.com (S.M.); 2 Nikolai Matveevich Kizhner Scientific and Educational Center, Tomsk Polytechnic University, Lenin Avenue, 30, Tomsk 634050, Russia; tvv@tpu.ru; 3 Department of Chemical and Biochemical Engineering, Satbayev University, Almaty 050013, Kazakhstan; g.aitkaliyeva@satbayev.university

**Keywords:** industrial waste, metallurgical slags, fly ash, clay, construction ceramics, composite materials

## Abstract

The growing accumulation of industrial waste and the depletion of natural mineral resources underscore the need for sustainable approaches to producing ceramic and construction materials. Among the most promising secondary raw materials are coal combustion by-products and metallurgical slags, which are suitable for ceramic applications. This review summarizes recent advances in the use of coal ash, blast furnace and steelmaking slags, together with clay-based raw materials, for the fabrication of ceramic and composite materials. Special attention is given to the physicochemical properties of technogenic raw materials and their effects on sintering, porosity, densification, mechanical strength, and thermal stability. Modern processing methods, including pressing and high-temperature firing, are also discussed. The influence of key technological parameters, such as oxide composition, particle size distribution, firing temperature, and activation conditions, is analyzed. In addition, the review examines major challenges related to raw material heterogeneity, structural instability, thermal stress development, cracking, free CaO reactivity, and environmental risks associated with heavy metal leaching. Recent studies show that incorporating industrial waste into ceramic systems reduces waste disposal, natural resource consumption, energy use, and CO_2_ emissions, while promoting sustainable and resource-efficient technologies. Ash- and slag-based ceramics therefore remain highly promising materials for construction applications.

## 1. Introduction

The construction industry consumes a significant portion of natural resources [1]; therefore, the rational use of secondary resources and the reduction in dependence on primary raw materials are of particular importance. Under intensive industrial and economic growth, consumption of natural resources increases significantly, which is accompanied by the accumulation of significant amounts of solid industrial waste [2,3].

Despite the need to dispose of solid industrial waste, in practice, burial remains the main method of its treatment. In some cases, these wastes are used in construction and agriculture, but such areas are classified as low-value uses and do not ensure the full realization of their resource potential [4].

According to a recent global assessment, about 2.01 billion tons of solid waste is generated annually worldwide, and forecasts indicate that by 2050 this volume could reach 3.4 billion tons. However, only about 33% of waste is properly disposed of, while the rest ends up in illegal or poorly controlled landfills. The waste management industry is undergoing significant transformations aimed at achieving sustainability and profitability in solid waste management, which could lead to increased operational efficiency and more sustainable waste management practices [5,6]. Improper handling of industrial waste and the lack of proper separation can mix waste streams, generating hazardous waste [7,8].

Proper waste management has become an urgent global priority. The problem of waste disposal and recycling remains particularly acute in developing countries, where effective waste management systems have not yet been formed [9]. In many countries, waste recycling is considered the main alternative to disposal, which reduces the negative impact on the environment and lowers the volume of solid waste [10]. Improper waste management practices lead to pollution, habitat destruction, and health risks, while effective systems can lead to significant economic benefits, including job creation, resource recovery, and reduced environmental restoration costs [11]. Therefore, further development of waste recycling and redirection methods is extremely important to avoid creating additional landfills that can significantly affect biodiversity and the environment [12].

The incorporation of industrial by-products, such as ash and slag, contributes to lowering CO_2_ emissions while enhancing the durability of materials [13,14].

However, despite extensive research on coal ash and metallurgical slags, several key challenges remain insufficiently understood. In particular, variability in raw material composition, differences in processing technologies, and the absence of well-established structure–property relationships result in considerable inconsistencies in the reported mechanical and functional performance.

In this review, industrial waste is analyzed from the perspective of its use as components for the production of composite materials. This paper examines the main types of industrial waste generated in different production sectors. Coal ash and ash and slag mixtures formed during fuel combustion in thermal power plants are analyzed. Special attention is paid to metallurgical slags, which are a large-tonnage mineral waste from the metallurgical industry.

A key priority for future development is the implementation of scientifically grounded, integrated, and resource-efficient technologies for the processing of anthropogenic waste. These approaches are aimed at transforming waste from landfill burdens into valuable secondary raw materials for the production of composite materials with tailored properties, thereby enhancing resource efficiency and reducing environmental impact.

## 2. Types of Industrial Waste

### 2.1. Coal Ash

#### 2.1.1. Definition and Chemical Composition

Coal ash is a finely dispersed industrial material generated during the combustion of coal in thermal power plants. It is collected from flue gases and represents one of the major solid by-products of the coal industry. The particles are typically spherical with smooth surfaces formed by high-temperature processes.

The chemical composition of coal ash is dominated by oxides such as SiO_2_, Al_2_O_3_, CaO, Na_2_O, Fe_2_O_3_, MgO, and K_2_O, along with trace elements including Cd, Cr, Pb, and Hg [15,16].

Table 1 presents compositional ranges reflecting variability observed across different studies and analytical techniques. These data highlight the differences between fly ash, bulk coal ash, and specific geochemical fractions. Such comparisons provide valuable insight into compositional trends and serve as a basis for the validation and calibration of predictive models for ash chemistry.

Coal combustion ash is commonly classified into Class C and Class F based on its chemical composition, mineralogical characteristics, and the type of parent coal. Class C ash, derived from lignite and sub-bituminous coal, is characterized by a CaO content exceeding 18% and contains a higher proportion of crystalline phases with relatively lower SiO_2_ content. In contrast, Class F ash originates from bituminous and anthracite coal, contains less than 18% CaO, and is enriched in SiO_2_, Al_2_O_3_, and Fe_2_O_3_.

**Figure 1:**
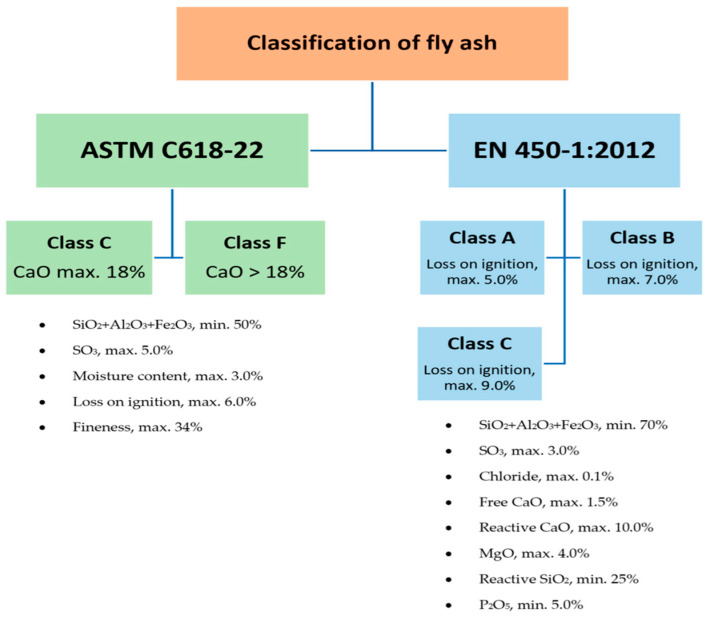
Classification of fly ash [27].

In European standards [25,26], coal ash is classified into Classes A, B, and C, and further categorized according to fineness as N (maximum residue of 40%) and S (maximum residue of 12%). Compliance with these classification criteria is illustrated in Figure 1 [27].

Ash generated during coal combustion is primarily produced in coal-fired power plants and is commonly referred to as coal combustion residues. Several types of ash are distinguished depending on their formation conditions and collection methods.

Fly ash is a fine particulate material, predominantly composed of silica, formed during the combustion of pulverized coal and transported with flue gases. Bottom ash, in contrast, consists of coarse, angular particles that accumulate at the bottom of the furnace due to their larger size [28]. Boiler slag (furnace ash) is produced directly within the combustion chamber, while flue gas desulfurization (FGD) residues result from processes used to remove sulfur oxides, enabling the reduction of up to 99% of SO_2_ emissions in conventional coal-fired power plants [29].

In general, ash and slag are products of thermal and phase transformations of the mineral fraction of coal and also contain minerals originally present in the parent rock [30].

SEM images (a–d) show that at elevated temperatures (>1400 °C), melting leads to the formation of predominantly spherical particles, whereas at lower temperatures (<900 °C, fluidized bed combustion (FBC), particles exhibit angular and irregular morphologies [31].

X-ray phase analysis (e) indicates the presence of quartz, mullite, gypsum, and other phases, with their relative proportions depending on the formation conditions. Fly ash is enriched in quartz and mullite, while bottom ash contains a higher fraction of the amorphous phase [31].

**Figure 2:**
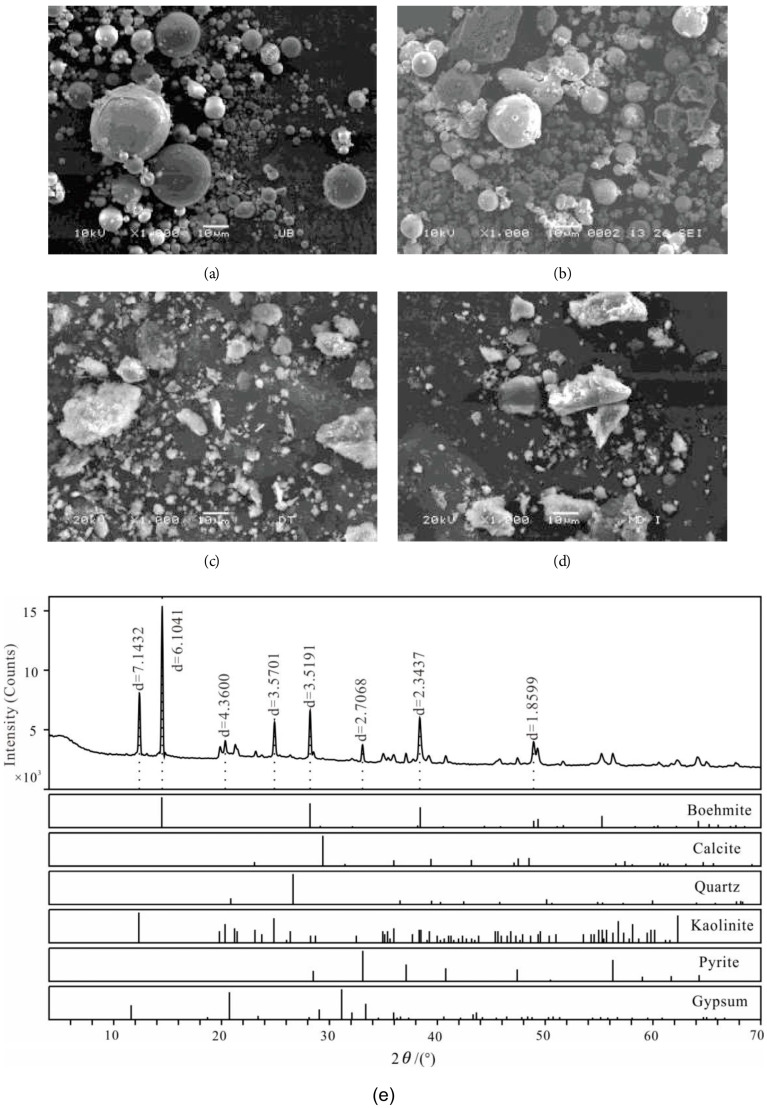
Morphological characteristics of fly ash samples and phase composition analysis of feed coal: (**a**) Scanning electron microscopy image of Uong Bi PCC fly ash [31]; (**b**) Scanning electron microscopy image of Ninh Binh PCC fly ash [31]; (**c**) Scanning electron microscopy image of Dong Trieu FBC fly ash [31]; (**d**) Scanning electron microscopy image of Mong Duong I FBC fly ash [31]; (**e**) XRD spectrum of feed coal from the Jungar Energy Gangue Power Plant [32].

Overall, combustion conditions play a key role in determining the structure and phase composition of the ash, thereby influencing its properties (Figure 2) [32].

Guoqiang Wu et al. (2024) [33] investigated coal samples from several thermal power plants using proximate and ultimate analyses (Table 2). According to the MT/T 849-2000 [34] classification, coal from the P4 power plant is characterized by medium to high volatile content (28.00–37.00%), whereas coal from the other plants exhibits high volatile content (37.00–50%). Based on the GB/T 15224.1-2018 standard [35], coals from P1, P2, and P3 are classified as low-ash (10.00–20.00%), while P4 coal falls into the medium-ash category (20.00–30%) [33].

Coal ash exhibits pozzolanic properties due to its siliceous or silico-aluminous composition. It consists of three main components: residual organic matter, an inorganic phase comprising both amorphous and crystalline structures, and minor liquid inclusions. Elevated carbon content in ash indicates incomplete combustion and limits its practical applicability [36].

The specific density of fly ash typically ranges from 1.9 to 2.2 g/cm^3^, whereas bulk coal ash density varies from 1.6 to 3.1 g/cm^3^. These values are strongly influenced by iron and carbon content: higher iron content increases density, while higher carbon content reduces it. Additionally, density is affected by particle size distribution, coal type, and morphology. The presence of hollow spherical particles, known as cenospheres, contributes to lower density and is advantageous in construction applications, as it reduces load and improves workability [37].

The properties of fly ash are determined by the characteristics of the parent coal, combustion technology, and collection systems (e.g., electrostatic precipitators) [38,39,40,41].

Coal ash strongly influences the phase formation and microstructure of ceramic materials because of its high content of amorphous SiO_2_ and Al_2_O_3_, as well as the presence of a glassy aluminosilicate phase. During heat treatment, the amorphous constituents exhibit high reactivity, actively participating in liquid-phase sintering and the formation of new crystalline phases.

One of the most significant effects is the enhanced formation of mullite (3Al_2_O_3_·2SiO_2_). The mullite phase is formed through the interaction between silica and aluminum oxide contained in the ash. Studies have shown that increasing the firing temperature promotes a higher mullite content and the development of needle-shaped or prismatic mullite crystals, which in turn improve the thermal resistance and mechanical strength of the ceramics.

The amorphous glassy phase of the ash plays an especially important role. At temperatures exceeding 1000–1100 °C, partial melting of the aluminosilicate components takes place, leading to the formation of a liquid phase that accelerates material sintering and promotes structural densification. As a result, open porosity is reduced, while the density and mechanical strength of the ceramic material increase [42].

In addition, increasing the temperature causes partial dissolution of crystalline quartz into the glassy phase. Infrared (IR) and Raman spectroscopy studies have demonstrated that at temperatures above 1100 °C, the quartz phase gradually transforms into an amorphous SiO_2_ state, accompanied by an increase in mullite content [43].

The presence of residual carbon and organic inclusions can also influence pore formation. During the burnout of organic components, additional pores are generated, which in moderate amounts contribute to lower density and the development of a lightweight structure. However, excessive amounts of unburned carbon may negatively affect the uniformity and mechanical properties of the material [15].

Coal ash can have long-term environmental impacts due to its weathering behavior and the potential release of contaminants. Therefore, modern waste management regulations require ash disposal in engineered containment systems to prevent leaching of hazardous components [44].

The reviewed studies demonstrate that the properties of coal ash depend on combustion conditions, particle size, and mineral composition. Its chemical composition is primarily governed by oxides of silicon, aluminum, iron, and calcium, along with trace elements whose concentrations depend on the source coal. Various classification systems (e.g., Class F and Class C, FA and BA types) reflect these differences, along with variations in physicochemical and functional properties.

Thus, the incorporation of coal ash results in several structural modifications in ceramics, including an increase in mullite phase content, a greater proportion of the glassy phase, a reduction in free quartz content, intensified liquid-phase sintering, decreased porosity, increased density, and enhanced thermal resistance and mechanical strength [15,42,43].

Despite its significant resource potential—particularly due to its high SiO_2_ and Al_2_O_3_ content—coal ash is still predominantly treated as waste. This is mainly due to concerns related to toxic elements and the need for controlled storage, which limits its broader utilization [45].

#### 2.1.2. Fly Ash Waste for Ceramics

The incorporation of coal ash into the production of environmentally sustainable building materials requires comprehensive characterization to evaluate its suitability and mitigate potential environmental risks [46]. Numerous studies have demonstrated that, with appropriate processing, coal ash can be effectively stabilized and exhibit hydraulic activity, thereby ensuring the long-term durability and environmental stability of the resulting materials after hydration [47].

The quantity and characteristics of waste generated during coal combustion are largely governed by the type of fuel (e.g., bituminous coal or lignite) and its mineralogical composition. Coal combustion residues represent a valuable source of mineral constituents, making them promising raw materials for the production of construction materials, particularly fired ceramics [48].

Alkali-activated coal ash is widely utilized as a primary component in ceramic tile production, contributing to the formation of phases such as quartz, hematite, mullite, and amorphous glass. Mechanical activation, including grinding, enhances the reactivity of ash and improves the sinterability of ash–clay mixtures [49]. According to Vakalova et al. (2023), semi-dry pressed fly ash samples without additives (FA100), fired at temperatures of 1100–1200 °C, exhibited relatively high porosity (water absorption of 19–26%) and comparatively low compressive strength (3–37.9 MPa), depending on the firing conditions [50].

**Figure 3:**
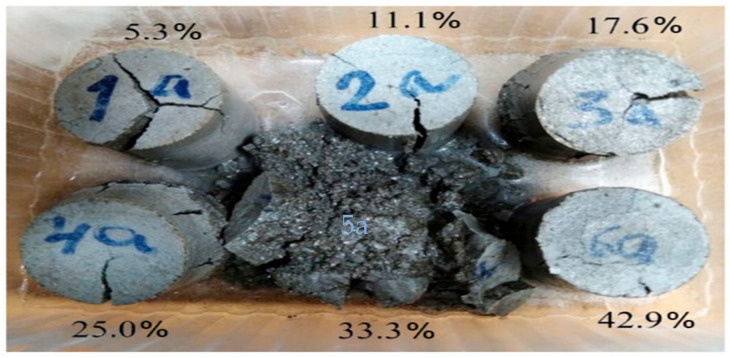
Expansion of specimens with silica fume additive contents of 5.3% (1a), 11.1% (2a), 17.6% (3a), 25.0% (4a), 33.3% (5a), and 42.9% (6a) by weight of fly ash after 11 days of air curing and 2 days of water curing [51].

X-ray diffraction analysis indicates that free calcium oxide (CaO) is present in silica-containing systems. The phase relationships between Ca(OH)_2_, CaO, and calcium hydrosilicates vary with silica content, as illustrated in Figure 3 and Table 3 [51,52,53]. The addition of microsilica (5.3–42.9 wt.%) significantly influences the expansion behavior of samples, particularly after combined curing in air (11 days) and water (2 days) [51,52,53].

Coal ash predominantly consists of SiO_2_, Al_2_O_3_, iron oxides, and varying amounts of alkali and alkaline earth metal oxides. These components play a critical role in determining the pore structure, phase composition, and physicochemical properties of ceramic materials.

Recent studies on porous ceramic composites based on silica–manganese slag and coal ash, with silicon carbide (SiC) as a pore-forming agent, have demonstrated the feasibility of producing materials with a highly porous structure. The processing route typically involves homogenization of raw materials with water, shaping (pressing), drying, and sintering at temperatures of 1050–1170 °C for approximately 45 min at a heating rate of 5 K·min^−1^ [54].

**Figure 4:**
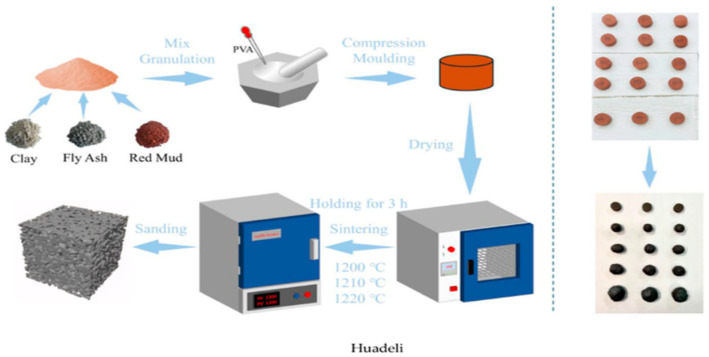
The preparation schematic diagram of foamed ceramics [57].

The technological workflow, schematically presented in Figure 4, includes raw material proportioning (Table 4), mixing of ash, red mud, and clay, followed by shaping and thermal treatment [55]. The mass of specimens is determined using high-precision analytical balances (accuracy of 10^−4^ g), while microstructural characterization is performed using scanning electron microscopy (SEM) [54]. The geopolymerization process proceeds through several critical stages that govern the development of the final porous structure [56].

Weng et al. (2025) proposed an optimized fabrication route involving the addition of polyvinyl alcohol (PVA) and silicon carbide (SiC), followed by pressing (15 mm diameter, 10 MPa, 90 s), drying at 100 °C for 6 h, and sintering at 1200–1220 °C for 3 h with natural cooling [57].

Despite these advancements, a major limitation in the application of coal ash in ceramic production is the variability in its chemical and phase composition. This heterogeneity can lead to non-uniform microstructures, increased water absorption, and reduced mechanical strength, thereby affecting the overall performance of the final products.

#### 2.1.3. Calculation Methods and Modern Research in Geopolymerization Processes

Mechanical strength and porosity were evaluated using standardized testing procedures. The analytical relationships used to determine tensile strength, bulk density, and apparent porosity, as reported by Krishnaraj et al. (2021) [58], are presented below. During testing, an initial load of 500 g was applied and gradually increased until specimen failure occurred.

The flexural (coupling) strength was calculated according to Equation (1):

(1) $$f_{\omega }=\frac{F_{1}e_{1}+\;F_{2}e_{2}-\frac{2}{3}d(F_{1}+F_{2}+\frac{\omega }{4})}{Z}$$

where $Z=\frac{{bd}^{2}}{6}$; b is the average bed joint width (mm), d is the mean depth of the prism (mm), $e_{1}$ is the distance between the applied load and the tensile face of the prism (mm), $e_{2}$ is the distance to the center of gravity of the clamping system on the tensile face (mm), $F_{1}$ is the applied load (N), $F_{2}$ is the self-weight of the loading setup (N), and ω is the weight of the detached portion of the masonry specimen at failure (N) [58,59].

An increase in fly ash content (up to 50 wt.%) results in a gradual reduction in mechanical strength. Nevertheless, brick-based systems exhibited the highest compressive strength values after 14 and 28 days of curing, indicating their suitability as alternative construction materials [58].

In contrast, Weng et al. employed a high-temperature synthesis route, where structural evolution is governed by sintering and crystallization at 1200–1220 °C. This approach promotes the formation of more stable crystalline phases and enables control over the porous structure [60].

According to the evaluation of physical and mechanical properties reported by Yu et al. (2023) [61], the bulk density and apparent porosity of sintered samples were determined using the method described by Equations (2) and (3).

Prior to testing, the samples were dried to a constant mass and weighed to obtain the dry weight (m_1_). The specimens were then immersed in water, and the water was subsequently boiled for 2 h to ensure complete saturation. After boiling, the system was allowed to cool naturally to room temperature, during which the immersed mass (m_2_) was measured. Finally, the samples were removed, their surfaces were gently wiped with a damp cloth to eliminate excess water, and the saturated mass in air (m_3_) was recorded.

The total porosity was calculated using Equation (4), while the closed porosity was determined according to Equation (5):

(2) $$\rho_{b}=\frac{m_{1}}{\left(m_{3}-m_{2}\right)}$$

(3) $$V_{open}=\frac{\left(m_{3}-m_{1}\right)}{\left(m_{3}-m_{2}\right)}\times 100\%$$

(4) $$V_{p}=\left(1-\frac{\rho_{b}}{\rho_{t}}\right)\times 100\%$$

(5) $$P_{closed}=V_{p}-V_{open}$$

where $\rho_{b}$ is the bulk density, $V_{open}$ and $V_{p}$ are the apparent and total porosity, respectively, $P_{closed}$ is the closed porosity, and $\rho_{t}$ is the true density of the material [61].

Thus, the key distinction between geopolymerization and sintering lies in their mechanisms of structure formation: the former is governed by gel network development, whereas the latter is controlled by diffusion and phase transformation processes. Consequently, these mechanisms lead to different material properties: sintered systems exhibit greater thermal resistance and structural stability, while geopolymer materials require lower energy input but are more sensitive to variations in raw material composition [62].

With increasing temperature, the structure becomes denser and strength improves, particularly with the addition of metakaolin and the use of a potassium-based activator. However, the development of thermal stresses leads to crack formation [61,62,63,64].

**Figure 5:**
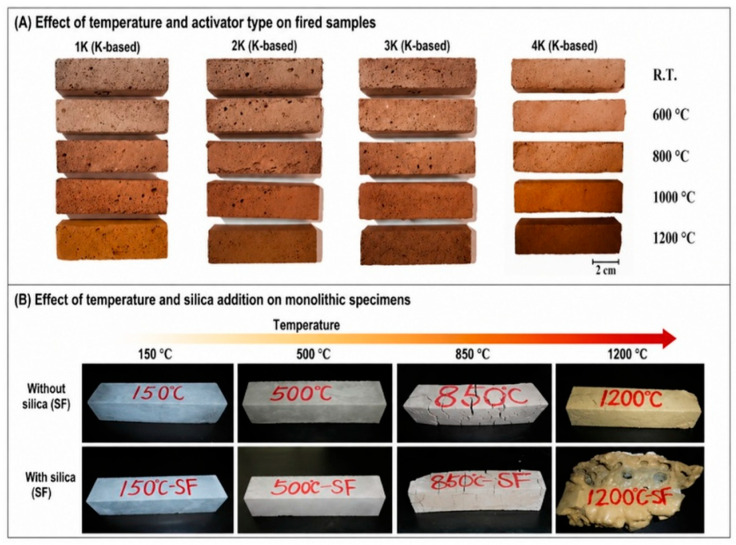
Effect of temperature and composition on the structure and thermal resistance of ash- and slag-based materials [63,65].

Silica-modified systems exhibit fewer cracks and maintain higher strength up to 850 °C. At 1200 °C, partial melting occurs, which diminishes the thermal resistance of the materials (Figure 5) [63,65].

A study by Yelubay et al. (2026) [66] demonstrates the feasibility of producing environmentally friendly, high-performance construction ceramics through the combined use of kaolinite clay, steel slag, and fly ash. Samples fired at 1100–1200 °C meet ASTM requirements for building bricks and tiles, with water absorption reduced to 3–5% and bulk density reaching up to 2.01 g·cm^−3^ [66].

Coal ash rich in SiO_2_, Al_2_O_3_, and CaO can partially replace natural clay in ceramic production without prior treatment. Hazardous components, including heavy metals and dioxins, are effectively immobilized within the amorphous glassy phase during high-temperature firing. Furthermore, blending ash with feldspar and other additives enables the production of ceramic materials with enhanced chemical resistance and mechanical strength. The presence of silicates, aluminosilicates, and quartz makes coal ash a valuable raw material for ceramic tile manufacturing. The alkaline activator solution was prepared from a liquid sodium silicate water glass (Betol 39 t) with a water glass modulus (SiO_2_/Na_2_O) of 3.20 and sodium hydroxide pellets. The solution was composed of 85.46 wt % liquid sodium silicate and 14.54 wt % sodium hydroxide, resulting in a water glass modulus of 1,28. The activator solution thus consisted of 23.44% SiO_2_, 18.37% Na_2_O and 57.90% H_2_O. To ensure consistent quality, each batch was homogenized for 24 h using a magnetic stirrer at a revolution per minutes of u = 500 rpm [67].

**Figure 6:**
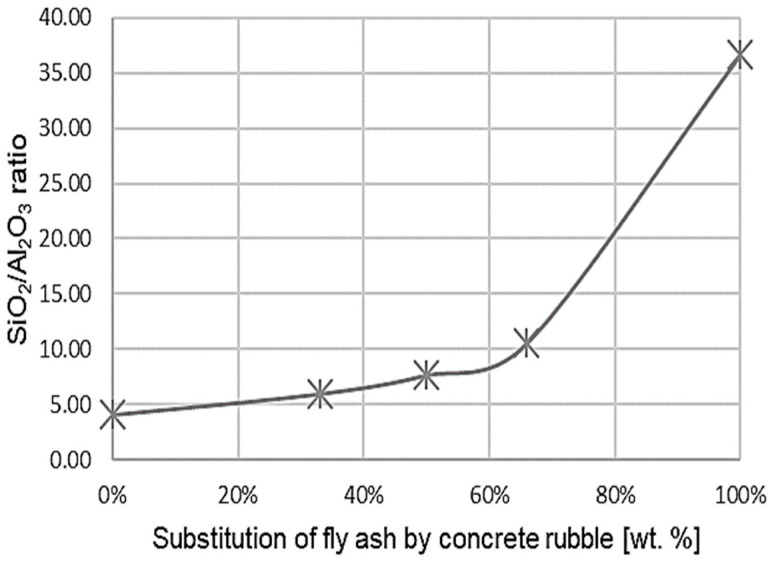
Mean values of compressive strength with increasing substitution of fly ash by concrete rubble [68].

Geopolymer systems incorporating 33 wt.% fly ash substitution achieved compressive strengths of up to 102.3 MPa, while a 50/50 mixture of fly ash and crushed stone reached 113.2 MPa. In contrast, geopolymers based solely on coal ash exhibited a strength of 83.2 MPa. The progressive increase in strength with higher incorporation of concrete rubble (Figure 6) is attributed to enhanced pozzolanic reactions and improved microstructural development [68].

Overall, coal ash is demonstrated to be a promising secondary raw material for ceramic production, contributing to the formation of key phases such as quartz, hematite, mullite, and amorphous glass. Mechanical activation (e.g., grinding) enhances its reactivity and improves sintering behavior in ash–clay systems. The reviewed studies also highlight key parameters influencing ceramic performance, including density, thermal expansion behavior, and processing conditions, as well as modern approaches for evaluating strength and frost resistance.

However, the utilization of coal ash is constrained by several limitations, including its inherent heterogeneity, high porosity, reduction in strength at high replacement levels, and sensitivity to elevated temperatures, which may lead to cracking and partial melting of the material.

### 2.2. Metallurgical Slags

#### 2.2.1. Metallurgical Slags and Their Chemical Composition

Metallurgical and steelmaking slags are classified according to the technological processes responsible for their formation. Blast furnace slags (BFSs) are generated during the reduction of iron oxides, pellets, agglomerates, and fluxes in the presence of coke, resulting in the production of molten iron in blast furnaces. In modern blast furnace operations processing ores containing 60–66 wt.% iron, approximately 200–300 kg of slag is produced per ton of molten metal [69,70].

The chemical composition of steelmaking slags, particularly Basic Oxygen Furnace (BOF) slag, is generally more variable than that of BFSs, reflecting differences in raw materials and processing conditions [71]. The investigated slag typically contains major oxides such as CaO, Fe_2_O_3_, SiO_2_, MgO, and Al_2_O_3_ and exhibits a complex phase composition. Its crystalline structure includes not only dicalcium and tricalcium silicates but also phases such as magnetite and brownmillerite [72].

**Figure 7:**
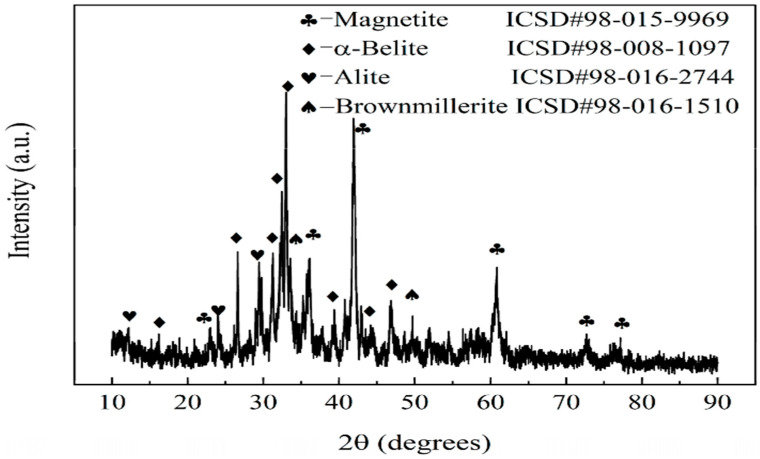
XRD results for the steel slag [72].

X-ray diffraction (XRD) analysis is widely used to identify the principal phase constituents of BOF slags. As illustrated in Figure 7, the diffraction pattern of the steel slag confirms the presence of multiple crystalline phases.

BFSs within the CaO–SiO_2_–Fe_2_O_3_–MgO–MnO system, particularly those with high basicity, may exist in heterogeneous states where liquid and solid phases coexist. Deviations between experimentally determined liquidus compositions and thermodynamic predictions have been reported, especially for MgO–CaO–SiO_2_ systems. In equilibrium conditions involving ferronickel and solid carbon, these discrepancies may reach approximately 4 wt.% MgO depending on temperature and CaO/SiO_2_ ratio [73].

Table 5 summarizes the compositional ranges of major oxides in blast furnace slags and related materials, including glass ceramics and silicate glasses. These data highlight the typically high CaO and SiO_2_ contents, along with variability in Al_2_O_3_ and Fe_2_O_3_ concentrations, which are governed by production parameters. Such variability is critical when evaluating slags as alternative raw materials for construction and materials science applications.

The dissolution of SiO_2_ in molten BOF slag significantly alters melt composition, influencing both phase equilibria and viscosity. Since viscosity strongly affects mass transfer processes, understanding these transformations is essential for predicting slag behavior during processing [80]. In blast furnace operation, maintaining the melt in a fully liquid state is crucial, as the presence of solid phases increases viscosity and disrupts process stability.

The liquidus temperature of cast iron (a carbon-containing alloy) is typically below 1200 °C, which is significantly lower than that of blast furnace slag. Therefore, maintaining slag temperatures above its liquidus point is essential to prevent premature crystallization and ensure efficient separation from the metal phase.

**Figure 8:**
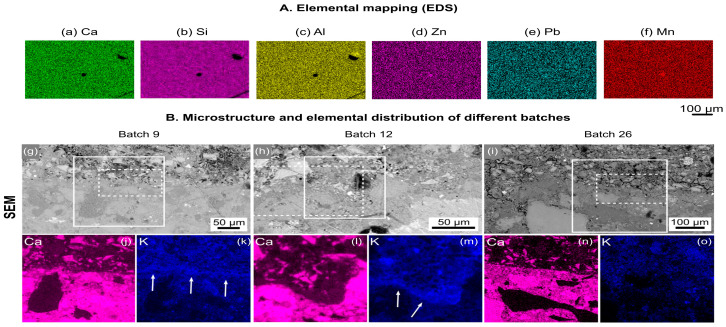
Comparative analysis of microstructure and elemental distribution in ceramic materials. (**A**): (**a**) EDS mapping of Ca; (**b**) EDS mapping of Si; (**c**) EDS mapping of Al; (**d**) EDS mapping of Zn; (**e**) EDS mapping of Pb; (**f**) EDS mapping of Mn [82]. (**B**): (**g**) SEM image of batch 9; (**h**) SEM image of batch 12; (**i**) SEM image of batch 26; (**j**) Ca elemental distribution map of batch 9; (**k**) K elemental distribution map of batch 9; (**l**) Ca elemental distribution map of batch 12; (**m**) K elemental distribution map of batch 12; (**n**) Ca elemental distribution map of batch 26; (**o**) K elemental distribution map of batch 26 [83]. White dashed-line boxes indicate the selected regions for detailed elemental mapping analysis. White solid-line boxes highlight the areas of interest, and arrows indicate the locations of K-enriched regions.

Calcium is linked to enhanced compaction, whereas potassium reflects incomplete homogenization [81]. Batch 9 exhibits a denser and more homogeneous microstructure. In contrast, batch 12 displays localized heterogeneity, while batch 26 is characterized by higher porosity and a lower degree of densification [82,83,84,85]. Overall, the composition has a pronounced influence on both the structure and the resulting properties (Figure 8).

The initial composition of the slag and the corresponding hot residue was determined based on averaged experimental data, enabling a reliable evaluation of their chemical and mineralogical characteristics, as well as their behavior during melting. The calculations included estimation of the mass of the hot residue, the initial slag mass, and the onset temperature of melting. It was established that slag composition is primarily governed by the conditions of direct iron reduction occurring in the shaft furnace, where iron ore interacts with reducing gases such as CO and H_2_.

Careful control of temperature and heating rates enables regulation of BOF slag behavior, ensuring efficient melting, phase stability, and separation from molten metal. Under optimized conditions, complete melting of metallic components is achieved while maintaining controlled slag formation, thereby enhancing process efficiency and the quality of the final material [86,87].

**Figure 9:**
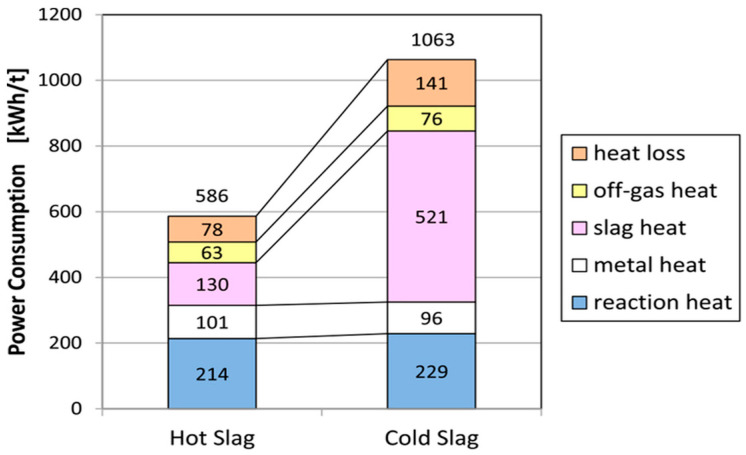
Comparison of required power consumption in each case of hot slag and cold slag charging [88].

The use of hot slag as a feedstock significantly reduces energy consumption. For instance, energy demand decreases from approximately 1063 kWh/t for cold slag to 586 kWh/t when hot slag is directly utilized, demonstrating substantial improvements in energy efficiency and processing time (Figure 9) [88].

In addition, increasing temperature induces the transformation of gehlenite into anorthite, a process typical of crystallization during heat treatment, as reported by Aziz et al. (2020) [89]. This observation underscores the crucial role of temperature in governing microstructural evolution and, consequently, the material’s properties [89].

Enhancing the utilization rate of steel slag while minimizing its environmental impact is essential for ensuring environmental safety, as well as the sustainable and stable development of the metallurgical industry [90].

This study provides a comprehensive characterization of steelmaking and blast furnace slags, emphasizing their key properties and distinguishing features. Particular attention is given to their chemical composition, which varies depending on raw materials and processing technologies, as well as to particle size and distribution, both of which significantly influence their potential applications.

Detailed analysis of the mineralogical composition, including the identification of major crystalline phases and the degree of amorphous content, is presented. Furthermore, X-ray diffraction data and microstructural observations are used to elucidate the structural and morphological differences among various slag types. The environmental performance of these materials is also evaluated, including the assessment of potential leaching behavior and associated ecological impacts.

Despite their considerable resource potential, the large volumes of metallurgical slags generated, combined with their complex multiphase nature, result in relatively low utilization rates and pose environmental challenges during storage and processing.

#### 2.2.2. Metallurgical Slags Waste for Ceramics

Metallurgical waste, traditionally regarded as a by-product requiring disposal, is now increasingly recognized as a valuable resource for the production of construction materials, including ceramics and concrete. Metallurgical slags formed during smelting processes exhibit advantageous properties such as high strength, durability, and thermal resistance [91].

When incorporated into construction materials, blast furnace slags can act as active components, forming silicates and oxides upon heating that contribute to strength development and reduced setting time [92,93]. Their performance is strongly influenced by chemical and mineralogical composition, glass-forming ability, and particle size distribution. The specific surface area, typically optimal within the range of 550–650 kg/m^2^, plays a key role in hydration kinetics, early strength development, and water demand. However, excessive grinding may adversely affect shrinkage, cracking, and setting behavior [94].

Ferrochrome slag materials were investigated by Marjaana Karhu et al. (2020) [95] using a combination of microstructural, compositional, and thermal analysis techniques. The results indicated that at 1000 °C, ceramic bonding is not fully developed, whereas at 1200 °C, well-defined ceramic bonds are formed, leading to the maximum strength of refractory concretes [95].

In asphalt systems, the incorporation of steelmaking slag has been reported to maintain a standard heating time while extending the cooling period. This prolonged cooling enhances the self-healing capacity of asphalt concrete, which is attributed to the physicochemical properties of steelmaking slag [96].

**Figure 10:**
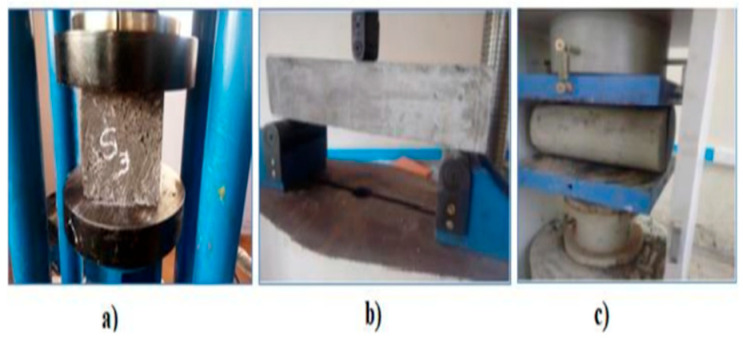
(**a**) Compressive test, (**b**) flexural test, (**c**) split tensile test of 28 days cured concrete specimen [97].

Mechanical testing was conducted using compression and universal testing machines with load capacities of 100 and 200 tons, respectively (Figure 10). The results demonstrate that the use of steelmaking slag as a coarse aggregate improves both the strength and durability of concrete, provided that the sulfate content remains below 0.5% and water absorption does not exceed 10%. Additionally, the presence of calcium oxide (CaO) in slag contributes to its partial binding activity, thereby enhancing the structural integrity and load-bearing capacity of the concrete matrix.

As shown in Table 6, both workability and mechanical strength of concrete are significantly improved when up to 75% of natural coarse aggregate is replaced with steelmaking slag [97].

Key parameters of building materials, including compressive strength and deformation behavior, play a fundamental role in structural design and modeling [98]. The higher ultimate flexural strength observed in these samples can be attributed to the superior impact resistance of ceramic coarse aggregates (27%) compared to natural aggregates (24%). Since impact resistance reflects the ability of aggregates to withstand sudden dynamic loads, it directly influences the overall strength performance of concrete [99].

A comprehensive understanding of the distribution and statistical characteristics of fibers within concrete is essential for both numerical modeling and practical structural applications. Fiber reinforcement significantly affects critical material properties, including mechanical strength, crack resistance, and post-peak behavior, thereby enhancing the toughness and durability of composite systems [100].

The expansion of infrastructure is closely linked to the increasing demand for cement, a key component of concrete which provides its mechanical strength. However, cement production is associated with substantial CO_2_ emissions, raising environmental concerns and emphasizing the need for more sustainable alternatives [101].

In addition to compressive strength and water absorption, several other factors critically influence the practical performance of building materials. These include wear resistance, thermal conductivity, frost resistance, and chemical stability, all of which must be considered for long-term durability and serviceability [102].

**Figure 11:**
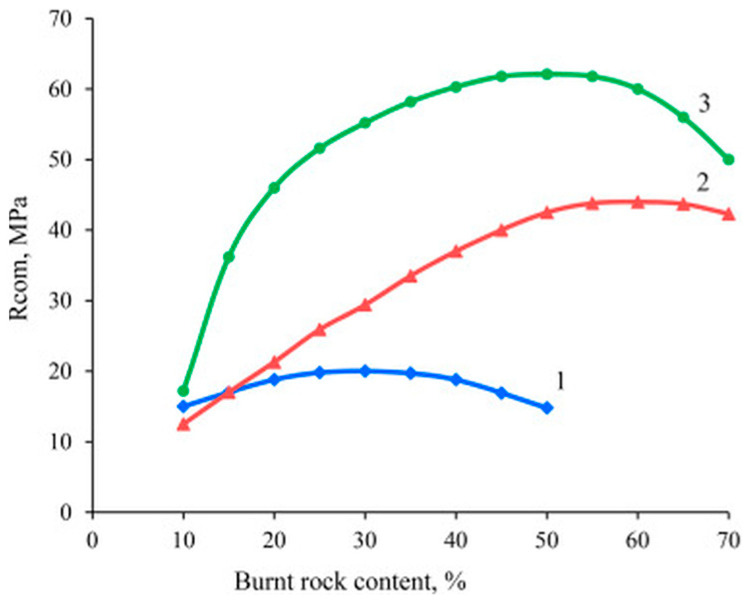
Influence of the content of burnt rock on the strength of the ceramic mass: 1—brick; 2—refractories; 3—tiles [103].

One of the key parameters governing the physicochemical and mechanical properties of ceramic bodies is the proportion of non-plastic components in the raw mix. As illustrated in Figure 11, the content of burnt rock significantly affects the strength of ceramic materials depending on the product type. For compositions based on refractory clays, the optimal burnt rock content ranges from 30 to 70%, whereas for conventional brick production, maximum strength is achieved at 20–40% burnt rock content [103].

**Figure 12:**
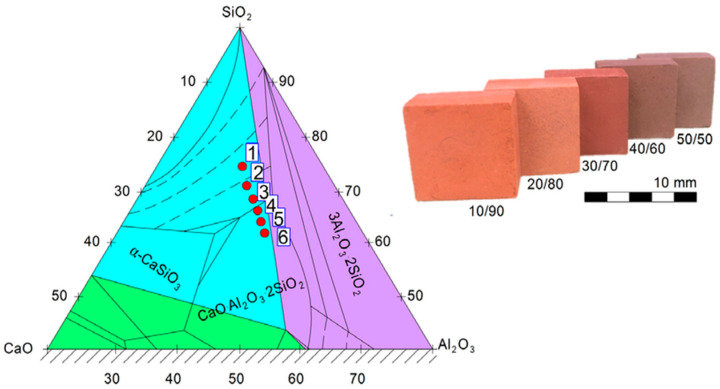
Ternary phase diagram of SiO_2_–Al_2_O_3_–CaO system. Points 1–5 correspond to ceramic compositions containing 10/90, 20/80, 30/70, 40/60, and 50/50 wt.% blast furnace slag/clay, respectively. Red circles indicate the compositions of the investigated mixtures, and the red line shows the compositional trend. The colored regions indicate different phase fields in the SiO_2_–Al_2_O_3_–CaO system [104].

In the study by Skripnikova et al. (2023), the influence of blast furnace slag content on phase formation in clay-based ceramic systems was systematically investigated (Figure 12) [104]. Samples prepared by semi-dry pressing followed by firing were used to construct a ternary phase diagram of the SiO_2_–Al_2_O_3_–CaO system. The compositions correspond to those presented in Table 7, covering a blast furnace slag content range from 0 to 50 wt.%.

The results demonstrate that the incorporation of blast furnace slag has a pronounced effect on the phase composition of the ceramics. In particular, the highest likelihood of anorthite crystallization was observed at a slag content of approximately 25 wt.%. This finding highlights the active role of calcium-rich slag components in promoting phase formation, thereby contributing to the development of the ceramic structure and the optimization of material properties [105].

Thus, unlike the approach of Skripnikova et al. [104], which primarily emphasizes phase equilibria in the SiO_2_–Al_2_O_3_–CaO system, the findings of Jamil et al. [105] demonstrate that structure development is governed by a complex interplay of chemical composition, activation parameters, and heat treatment conditions.

This highlights the limitations of relying solely on phase relationships, without considering process kinetics and activation conditions [105].

Modern construction and advanced technical ceramics differ substantially from conventional materials, as their properties are largely governed by the deliberate engineering of chemical composition and microstructure. In this context, the incorporation of industrial waste as functional components is of particular interest, as it enables controlled phase formation and the design of materials with tailored performance characteristics.

Mangialardi et al. (2021) conducted an experimental investigation on the production of single-component alkali-activated materials derived from industrial by-products, including granulated blast furnace slag and spent sands from biomass boilers [106]. The synthesized materials exhibited high compressive strength, demonstrating their suitability for structural applications. Furthermore, their utilization contributes to reducing the volume of industrial waste and mitigating its environmental impact [106,107,108,109,110].

**Figure 13:**
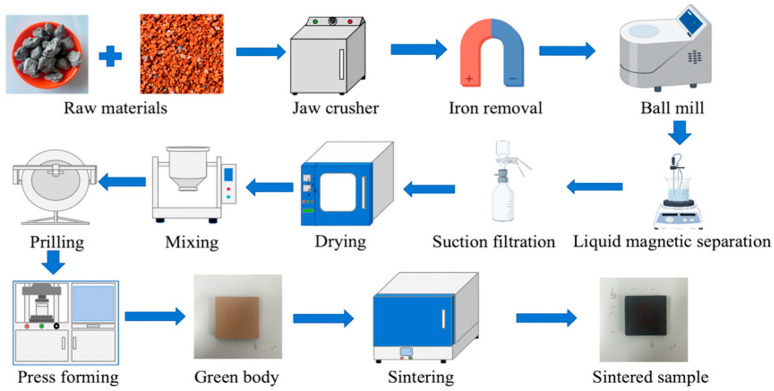
Schematic diagram of the sintered tile preparation process [111].

Similarly, Ji et al. (2024) [111] explored the fabrication of ceramic materials based on steel slag, waste clay bricks, and talc. The processing route involved raw material grinding, homogenization, shaping by pressing, followed by drying and high-temperature sintering (Figure 13) [111]. The results indicate that the use of such composite formulations promotes the formation of a dense ceramic microstructure and enables the production of sintered tiles with enhanced physicomechanical properties. These findings highlight the strong potential of metallurgical and construction wastes as alternative raw materials in ceramic manufacturing [111,112].

The Al_2_O_3_ content plays a crucial role in the structural evolution of ceramic materials. On the one hand, alumina promotes phase formation and crystallization processes; on the other hand, it acts as an intermediate network-forming component capable of substituting Si^4+^ within the tetrahedral framework (AlO_4_ units), thereby contributing to the development of glassy networks and crystalline phases. However, the presence of octahedrally coordinated aluminum (AlO_6_) may destabilize the structure, leading to a disruption of its long-range order.

As the Al_2_O_3_ content increases, the proportion of Si^4+^ within the network structure decreases, while the formation of AlO_6_ octahedral units further contributes to structural depolymerization. The key reactions governing these processes can be expressed as follows [113]:

(6) $$CaO+SiO_{2}\to CaSiO_{3}$$

(7) $$CaO+{Al}_{2}O_{3}+SiO_{2}\to {Ca}_{2}{Al}_{2}SiO_{7}$$

In contrast to the findings of Ji et al. (2024) [111], where the use of slag- and construction waste-based compositions results in a dense microstructure and enhanced strength, Zhang et al. [114] demonstrated that the incorporation of carbonate-rich waste may produce the opposite effect. Specifically, increasing the proportion of such waste reduces strength due to dilution and a lower content of reactive components, despite the partial contribution of CaCO_3_ to the formation of C–A–S–H gel.

Therefore, unlike the conclusions of Ji et al. (2024) [111], the effectiveness of waste utilization in ceramic and geopolymer systems depends not only on its presence but also on its chemical composition and reactivity [114].

In the context of transitioning toward sustainable raw materials, the combined use of industrial waste and functional additives has gained significant attention for optimizing material compositions. Blast furnace slag serves as a primary component of the batch due to its high content of oxides (CaO, MgO, Al_2_O_3_, Fe_2_O_3_, SiO_2_), which promote both vitrification and the crystallization of pyroxene phases.

To tailor material properties, various additives such as coal ash, quartz sand, dolomite, limestone dust, and soda ash have been employed, as reported by Alexander V. Gorokhovsky et al. (2023) [115]. Coatings developed from blast furnace slag and electroplating sludge exhibit high impact resistance, strong adhesion to carbon steel, and compliance with GOST 24788-2018 standards [116]. Based on these components, raw material mixtures with different compositions were formulated, and the characteristics and proportions of the constituents used for glass formulations are summarized in Table 8 [115,117].

The steel industry generates approximately 600 kg of solid waste per ton of steel produced, including scale, sludge, and dust, many of which contain substantial amounts of iron. Although a large portion of this waste is still disposed of in landfills, a significant share can already be recycled internally or utilized as by-products in other industries. Mill scale is a residual material formed as a layer of iron oxides on the steel surface during continuous casting and rolling processes. It develops when steel is exposed to elevated temperatures in the presence of oxygen, which promotes oxide layer formation. The chemical composition of mill scale depends on the type of steel produced and the manufacturing process employed. Typically, it contains around 70% iron, along with minor quantities of non-ferrous metals and alkaline compounds. However, only a portion of the generated scale can be reused in the sintering process at integrated steel plants [118,119].

Based on the experimental results reported by Siriwattanakarn et al. (2025), it can be concluded that the use of mill scale waste as a natural fine aggregate resulted in reduced workability and thermal conductivity, whereas porosity and dry-state density showed a slight increase [118].

The incorporation of mill scale waste into construction mixtures has demonstrated several effects, including reduced workability and thermal conductivity, along with a slight increase in porosity. At the same time, it improved chloride resistance and helped preserve residual compressive strength even after exposure to elevated temperatures.

These results suggest that the substitution of natural fine aggregate with mill scale waste represents an effective strategy for waste recycling and the conservation of natural aggregate resources in construction applications [118].

This section highlights the potential of metallurgical slags as promising secondary raw materials for the production of ceramics, composite materials, and high-performance concretes with enhanced strength and durability. It is demonstrated that such slags can be effectively incorporated into construction materials, contributing to improved physicomechanical properties and functional performance. The key components of raw material mixtures for ceramic fabrication are outlined, along with a generalized technological scheme for the production of sintered tiles. Particular emphasis is placed on the utilization of steelmaking slag in glass-ceramic systems, where it serves as an active component in composite formulations. The synergistic relationship between metallurgical slags and coal ash is also addressed.

Despite their advantageous properties, the application of metallurgical slags in ceramic systems remains constrained by the sensitivity of their performance to variations in chemical composition, particle size distribution, and processing conditions. These factors may lead to structural instability, crack formation, and deterioration of technological characteristics, thereby limiting their broader implementation.

## 3. Clay Raw Materials for Ceramic Products

Clay deposits represent complex multicomponent systems composed of various clay minerals with similar chemical compositions and crystal structures. These materials exhibit a wide range of performance characteristics, primarily governed by their plasticity and their ability to bind non-plastic additives [120].

According to Chenyang et al. (2025) [121], compaction tests were conducted using a mold with an inner diameter of 10 cm and a height of 12.7 cm. After thorough homogenization, the mixture was placed into the mold in three equal layers, each subjected to 27 blows. Upon completion, the specimen surface was carefully leveled using a knife and ruler to ensure flat and parallel upper and lower surfaces [121].

When interpreting experimental results, it is essential to consider the type of clay raw material, as this parameter significantly influences the classification of textured clays, ranging from light to heavy types [122]. Clays serve not only as the primary raw material but also as functional additives capable of regulating both technological and operational properties of ceramic systems. Their influence is determined by granulometric distribution, mineralogical composition, and chemical characteristics, which collectively affect plasticity, sinterability, and the final microstructure of the material.

As summarized in Table 9, clays play a critical functional role by ensuring formability, structural integrity, and control over sintering processes. Depending on their chemical composition (e.g., Al_2_O_3_, Fe_2_O_3_, and fluxing oxides) and mineralogical constituents (such as kaolinite, illite, and smectite), clays can enhance plasticity, adjust firing temperatures, and regulate the density and overall performance of ceramic materials.

Clay extraction requires a more controlled and selective approach than overburden removal, as both the quality and the degree of extraction directly influence the properties of the final product.

**Figure 14:**
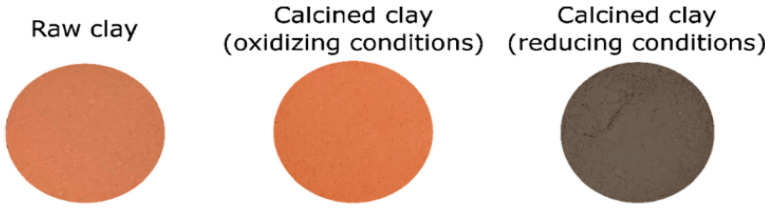
Color of raw and calcined kaolinitic clay under various firing conditions [128].

Figure 14 illustrates the color variation between raw and calcined kaolin clay containing 3.8 wt.% iron oxide when fired under oxidizing and reducing atmospheres [128]. Under reducing conditions, the calcined clay exhibits a gray coloration, whereas oxidative firing results in a pinkish-red hue. This difference is attributed to the formation of magnetite under reducing conditions, in contrast to hematite under oxidizing conditions.

Chemical interactions between clay minerals and various additives can significantly modify the physicochemical properties of the material, enhancing its stability. Clays are characterized by high plasticity and a pronounced tendency for volumetric changes in response to moisture fluctuations.

Control of the final color can be achieved primarily through two approaches: (i) rapid cooling (quenching), which inhibits oxidation to hematite during temperature reduction, and (ii) cooling in a reducing atmosphere, which limits oxygen availability and stabilizes reduced iron phases [129].

One of the most promising approaches for activating 2:1 type clays is mechanical or mechanochemical activation, which requires significantly lower energy input compared to conventional calcination. This process relies on the combined effects of impact forces and friction, leading to structural disruption, partial amorphization, and consequently enhanced chemical reactivity of the clay. In addition, mechanical activation results in particle size reduction and alterations in particle morphology. However, under high-energy treatment conditions, a decrease in specific surface area may occur due to particle agglomeration and the formation of new interparticle bonds. Overall, these structural and morphological modifications substantially increase the reactivity of clays, making them attractive as pozzolanic materials [130].

Clays are unconsolidated sedimentary materials or their lithified counterparts (claystones), composed predominantly of fine-grained silicate minerals. Kaolinite is a dioctahedral 1:1 layer silicate in which the octahedral sites are occupied exclusively by aluminum. Montmorillonite, a principal member of the smectite group, is characterized by a 2:1 layered aluminosilicate structure with a layer charge typically ranging from 0.2 to 0.6. This charge is mainly localized in the octahedral sheet, where Al^3+^ ions are partially substituted by divalent cations, primarily Mg^2+^. Illite is a 2:1 phyllosilicate structurally similar to muscovite and commonly formed through its weathering, although it allows for a wider range of cation substitutions. Muscovite itself is a well-ordered 2:1 phyllosilicate in which approximately one-quarter of the tetrahedral Si^4+^ ions are replaced by Al^3+^, resulting in a stable layered structure [131,132].

Ettringite is a highly hydrated crystalline phase formed through the reaction of soluble sulfates, calcium (typically derived from lime), and alumina in the presence of water. Within the pore structure, ettringite crystallizes as needle-like formations with a colloidal morphology. At early stages, its formation contributes to strength development by reducing porosity, creating interlocking crystal networks, and lowering moisture content through water uptake [133].

Regulatory frameworks governing ceramic production permit the incorporation of secondary raw materials and industrial by-products, including those originating from the construction sector [134].

From an industrial perspective, the Al_2_O_3_/Fe_2_O_3_ ratio serves as a key indicator for assessing the suitability of clays in ceramic formulations. Clays with an Al_2_O_3_/Fe_2_O_3_ ratio greater than 5.5 are alumina-rich, typically exhibit lighter coloration, and are well suited for refractory ceramic applications. In contrast, clays with a ratio below 5.5 contain higher iron content and are primarily used in the manufacture of building materials such as bricks, tiles, and related products [135].

Careful control of raw material composition is essential to produce ceramic tiles that meet both mechanical and aesthetic requirements. Ceramic tiles are commonly manufactured from a mixture of natural and synthetic components, including aluminosilicate clays, supplemented with minor additions of pigments, metal oxides, and colorants [136].

Natural clays exhibit considerable compositional variability, even within a single deposit. Clay minerals are classified based on the arrangement of tetrahedral and octahedral sheets into 1:1, 2:1, and 2:1:1 phyllosilicate structures. A representative example of 1:1 phyllosilicates is kaolinite, in which the external positioning of hydroxyl groups on octahedral sheets contributes to a relatively low optimal calcination temperature [137].

The incorporation of lime and nanozeolite into kaolinite-based systems has been shown to significantly enhance mechanical strength during long-term curing. For instance, the strength of kaolinite increased steadily from 250 to 685 kPa over a curing period of 360 days, followed by a slight decrease to 590 kPa after 720 days [134]. Increasing the curing temperature from 20 to 40 °C further promotes strength development due to the formation of calcium silicate hydrate (C–S–H) gels [138,139].

This section outlines the key characteristics of clay raw materials used in ceramic production, including classification approaches applied across different soil systems. Particular attention is given to features enabling differentiation between raw and calcined kaolin based on color. Various types of clay materials classified as non-hazardous waste and suitable for incorporation into ceramic mixtures are also described. The discussion highlights the role of clays in ceramic compositions, their morphological features, and phase composition, which collectively govern material behavior during shaping and firing.

Nevertheless, it should be noted that clay raw materials exhibit significant mineralogical and granulometric heterogeneity, along with high sensitivity to thermal treatment conditions. These factors complicate the achievement of consistent physico-mechanical properties in ceramic products.

## 4. Practical Limitations and Prospects

The utilization of industrial waste, including coal ash, metallurgical slags, and clay-based materials, in the production of ceramics and construction materials is associated with several practical challenges. A primary limitation is the significant variability in the chemical and phase composition of these raw materials, arising from differences in feedstock sources and processing conditions. This variability often results in inconsistencies in the physico-mechanical properties of the final products, such as strength, porosity, and water absorption. Additional constraints are linked to the presence of potentially hazardous constituents, including heavy metals and free CaO, which necessitate careful control and stabilization to ensure environmental safety [18,30,50,51,52,53,54,55,56].

From a technological perspective, the optimization of processing parameters presents further challenges. Factors such as particle size distribution, firing regimes, and batch composition must be carefully controlled. For instance, increasing the proportion of coal ash may lead to reduced mechanical strength and increased porosity, while excessive grinding of slags can promote shrinkage and cracking. Moreover, mismatches in the thermal expansion coefficients of individual components can generate internal stresses during high-temperature processing, resulting in structural defects [84,85,86,87,124,125,126].

Despite these limitations, industrial raw materials offer considerable potential for advanced material development. As summarized in Table 10, their properties, limitations, and application areas highlight opportunities for optimization. A particularly promising direction involves the design of composite systems with tailored chemical and mineralogical compositions. Approaches such as alkali activation and the incorporation of functional additives—including silica, metakaolin, and other modifiers—can enhance raw material reactivity, improve sintering behavior, and promote the formation of dense, mechanically robust, and structurally stable materials.

Another important direction involves the implementation of energy-efficient processing technologies, including the utilization of hot slags and the mechanochemical activation of clays, both of which contribute to reduced energy consumption and improved overall process efficiency. In addition, optimization of oxide ratios—particularly within SiO_2_–Al_2_O_3_–CaO systems—and precise control of phase formation enable the design of materials with tailored performance characteristics, such as enhanced mechanical strength, thermal stability, and long-term durability [70,71,72,73,74,75].

According to A. Kuanyshbay et al. (2025) [140], high-strength ceramic materials can be achieved by forming a fine-pored structure with densely packed pore walls, which reduces the likelihood of crack and defect formation. The coagulation capacity of clay raw materials plays a crucial role in determining the physical and technical characteristics of the final product, particularly its density, porosity, pore size, and pore distribution [140].

Recent studies in China highlight the rapid development of ceramic materials derived from industrial waste such as coal ash and metallurgical slags. For example, Chao Cheng et al. (2020) demonstrated the feasibility of producing porous ceramic membranes from ash with high porosity (~37%) and a controlled microstructure [141]. Yanbing Zong et al. (2018) reported that incorporating ash into slag-based systems promotes the formation of anorthite; however, excessive ash content reduces mechanical strength due to increased porosity [142].

Furthermore, Zhaohui He et al. (2025) showed that combining ash with electrolytic manganese residue enables the production of ceramic composites with enhanced density and firing strength at approximately 1150 °C [143]. Ash has also been effectively utilized in the synthesis of glass-ceramic materials, improving both density and chemical stability [144]. Nevertheless, the reported results remain inconsistent, largely due to variations in raw material composition and processing conditions [145].

Recent studies indicate that ceramic and composite materials derived from ash and slag possess considerable potential for practical applications. They are widely utilized in the manufacture of construction products such as bricks, ceramic tiles, lightweight aggregates, and refractory materials.

Ash-based ceramics are typically characterized by low density and good thermal insulation performance, making them attractive for energy-efficient construction. In contrast, systems incorporating metallurgical slags exhibit enhanced strength and durability due to the formation of calcium-rich phases.

Moreover, geopolymer and alkali-activated materials produced from ash and slag demonstrate high mechanical strength (up to 100 MPa or higher) along with excellent chemical resistance, supporting their application in advanced building ceramics and composites.

The construction industry plays an important role in waste recycling by utilizing waste materials directly as raw materials or additives. Nevertheless, the large volumes of waste still being disposed of remain a serious environmental concern. Fly ash is also regarded as a promising low-cost adsorbent material that can be used either directly or after functionalized synthesis. In addition, it is considered an alternative resource for the metallurgical extraction of high-purity products such as alumina [145].

Life Cycle Assessment (LCA) studies have demonstrated that the incorporation of fly ash and other industrial wastes into ceramic systems can significantly reduce environmental impacts compared with conventional ceramic production methods and landfill disposal practices (Table 11). The reuse of fly ash contributes to lower greenhouse gas emissions, conservation of natural resources, reduced landfill accumulation, and improved sustainability of ceramic manufacturing processes. In particular, lightweight aggregate systems based on fly ash exhibit lower global warming potential and reduced ecotoxicity relative to traditional disposal scenarios [146].

The practical value of these materials lies in their ability to enable large-scale utilization of industrial waste while producing competitive construction products. Their use also contributes to lower energy consumption and reduced CO_2_ emissions compared to conventional ceramic manufacturing, thereby decreasing environmental impact and supporting sustainable construction.

Therefore, overcoming existing limitations requires an integrated approach that combines detailed characterization of raw materials, optimization of processing parameters, and the application of advanced activation and modification strategies. Such an approach provides a solid foundation for the development of environmentally safe, high-performance ceramic and construction materials.

## 5. Conclusions

This review presents recent advances in the development of ceramic and composite materials based on coal ash and metallurgical slags. These industrial by-products show strong potential as secondary raw materials owing to their high content of aluminosilicate and calcium-rich phases, which promote the formation of robust and stable material structures.

Analysis of the literature indicates that the properties of the resulting materials are largely governed by raw material composition, synthesis parameters, and firing temperature. In particular, phase formation is identified as a key factor controlling the mechanical strength, thermal stability, and long-term durability of ceramic systems.

Despite existing limitations associated with compositional heterogeneity and the presence of potentially hazardous impurities, the findings confirm the effectiveness of an integrated approach to the utilization of industrial waste.

Thus, the utilization of ash and slag in the production of ceramic and composite materials represents an efficient approach that integrates enhanced material performance with reduced environmental impact, aligning with contemporary principles of sustainable development.

## Figures and Tables

**Table 1:** Comparison of measured and predicted concentrations of major elements in fly ash and coal ash samples.

Description of Analyzed Material	CaO (%)	Fe_2_O_3_ (%)	SiO_2_ (%)	MgO (%)	MnO (%)	Al_2_O_3_ (%)	TiO_2_ (%)	Cr_2_O_3_ (%)	Zn (%)	Refs.
Major oxides in coal fly ash	0.67–2.04	2.86–6.61	50.16–44.98	0.40–0.58	0.09–0.211	41.48–38.89	2.11–2.04	43.05–53.59	0.91–1.59	[17,18,19,20,21]
Geochemical composition of fly ash	0.8–4.15	4.2–5.63	11.3–28.1	0.38–0.41	0.04–0.08	4.8–8.23	0.8–0.57	17.8–100	0.88–0.70	[22]
Chemical composition of coal ash	2.11	2.29	72.10	0.76	95.99	19.22	1.31	67.84	17.39	[23]
composition of amorphous phases of fly ash	35.1	9.3	29.7	3.4	0.5	13.3	2.1	0.6	0.5	[24]

**Table 2:** Proximate and ultimate analysis results of coal samples (wt%) [33].

		Proximate Analysis				Ultimate Analysis			
Sample	M_ad_	V_daf_	A_d_	FC_d_	C_daf_	H_daf_	N_daf_	S_d_	O_daf_
P1-coal	6.36	37.79	15.65	52.48	72.34	5.62	1.24	0.40	26.90
P2-coal	5.83	37.48	18.06	51.23	89.54	5.59	1.53	0.48	11.91
P3-coal	4.98	37.76	15.28	52.74	84.65	5.15	0.97	0.71	14.16
P4-coal	4.05	37.30	20.60	51.37	84.97	16.46	2.58	1.12	11.72

M: moisture, V: volatile matter, A: ash yield, FC: fixed carbon, C: carbon, H: hydrogen, N: nitrogen, S: sulfur, O: oxygen, ad: air-dry basis, d: dry basis, daf: dry and ash-free basis.

**Table 3:** Crystalline phases of fly ash [51].

Sample Type	Phase Content (%)		
	Ca(OH)_2_	CaO_free_	Hydrosilicates
Fly ash original	-	100	-
Fly ash after hydration	96.2	-	3.8
Fly ash +11.1% silica fume after hydration	80.1	8.1	11.8
Fly ash +25% silica fume after hydration	67.8	17.9	14.3
Fly ash +42.8% silica fume after hydration	63.3	18.2	18.6

**Table 4:** Specimen formulations [57].

Number, X = A (1200 °C), B (1210 °C), C (1220 °C)	Fly Ash/wt.%	Red Mud/wt.%	Clay/wt.%	SiC (Addition)/wt.%	Binder Agent (Addition)/wt.%
X1	45	45	10	0	10
X2	45	45	10	0.5	10
X3	45	45	10	1.0	10
X4	45	45	10	1.5	10
X5	45	45	10	2.0	10

**Table 5:** Ranges of chemical compositions of different metallurgical slags.

Description of Analyzed Material	CaO (%)	Fe_2_O_3_ (%)	SiO_2_ (%)	MgO (%)	MnO (%)	Al_2_O_3_ (%)	TiO_2_ (%)	Cr_2_O_3_ (%)	SO_3_ (%)	Refs.
Blast furnace slag is primarily composed	30–50	10–40	28–38	1–18	5–8	8–24	3–5	2–5	3–5	[74]
Blast furnace slag-based glass ceramics	24.95–25.33	0.90–0.98	49.90–50.66	4.38–4.46	3.40–3.94	9.60–9.80	4.85–4.96	0.20–2.00	3.40–3.94	[75]
Oxide composition of glasses and blast furnace slag	23.89–39.81	2.73–3.52	31.92–48.19	5.12–8.54	3.10–4.85	9.07–16.07	1.85–3.40	2.73–4.10	3.20–3.52	[76,77,78]
blast furnace slag	36.79–50.74	0.34–1.48	25.59–38.21	6.13–9.96	0.13–0.9	11.38–15.88	0.84–6.71	0.02	25.59–38.21	[79]

**Table 6:** Mix proportions of M30 grade fresh concrete (kg/m^3^) [97].

Sample Name	Cement (kg)	Water (kg)	Fine Aggregate (kg)	Coarse Aggregate (kg)	Steel Slag (kg)	Remark
S1	387.5	186	680	1110	-	Control mixture
S2	387.5	186	680	832.5	277.5	25% replacement of coarse aggregate by steel slag
S3	387.5	186	680	555	555	50% replacement of coarse aggregate by steel slag
S4	387.5	186	680	277.5	832.5	75% replacement of coarse aggregate by steel slag
S5	387.5	186	680	0	1110	100% replacement of coarse aggregate by steel slag

**Table 7:** Raw mixture components for ceramic specimens produced in laboratory conditions [104].

Raw Mixture Components		Content, wt.%				
Clay	100	90	80	70	60	50
Blast furnace slag	0	10	20	30	40	50
Oxide ratios, a.u. *						
CaO/SiO_2_	-	0.122	0.131	0.141	0.153	0.166
CaO/Al_2_O_3_	-	0.491	0.493	0.495	0.497	0.499

* a.u. means arbitrary units.

**Table 8:** Raw material mixtures used to formulate glass composition [115].

Raw Material	Batch Number				
	1	2	3	4	5
Fly Ash	30	-	30	-	-
Limestone	38	-	-	-	-
Silica Sand	15	23	17	24	24
Galvani Slurry	7	12	7	13	14
Soda Ash	10	8	8	13	13
Dolomite dust	-	17	38	20	19
Slag	-	40	-	30	30

**Table 9:** The functional role of clay as an additional component in ceramic materials: influence on properties and operational characteristics.

	Summary Characteristics of Clay	Functional Properties	Clay Minerals as Additional Components	Refs.
1	Silty–clayey fractions, smectite, illite and kaolinite	high values of plasticity and moldability, efficient sintering, low shrinkage	Ceramic mass base, improves moldability and compaction	[123]
2	Raw clays containing 15–20% Al_2_O_3_, 3–9% Fe_2_O_3_, and at least 2% fluxing oxides	Control of strength, porosity, and firing temperature	Regulates the composition and properties (bricks, tiles, refractories)	[124]
3	Fine-grained structures (<2 μm), layered aluminosilicates	High surface, cation exchange capacity (CEC), porosity	Increases reactivity and interaction in the system	[125]
4	Kaolinite, illite, montmorillonite and others	Adsorption, plasticity, stability	A multifunctional component used across different materials and composites.	[126]
5	Clay pastes, dependence on water and grain size distribution	Rheology, plasticity, shrinkage	Provides moldability and stability of the structure (including AM)	[127]

**Table 10:** Properties, limitations, and applications of technogenic raw materials.

Material	Main Composition	Limitations	Processing Features	Applications	Refs.
Coal fly ash	SiO_2_, Al_2_O_3_, Fe_2_O_3_, CaO, trace heavy metals	High porosity, low strength, environmental risks	Grinding, alkali activation, sintering (1100–1200 °C), silica fume addition	Ceramic tiles, geopolymers, lightweight ceramics	[40,41,44,45,47]
Coal ash (general)	Silicates, aluminosilicates, CaO, Fe oxides	Composition variability, sensitivity to combustion conditions	Classification (Class F, C), particle size control	Cement, construction materials	[22,23,24,42]
Metallurgical slag	CaO–SiO_2_–Al_2_O_3_–Fe_2_O_3_–MgO system	Variable composition, heavy metals, melt viscosity	Cooling control, grinding, thermal processing	Cement, ceramics, glass-ceramics	[63,64,65,66]
Steelmaking slag	CaO-rich silicates, ferrites	Free CaO expansion, cracking risk	Fine grinding, controlled hydration	Concrete aggregates, composites	[83,84,85,86]
Slag-based glass-ceramics	CaO–SiO_2_–Al_2_O_3_ with modifiers	Composition sensitivity	Sintering (1000–1200 °C), flux addition	Glass-ceramic coatings, tiles	[96,104,105]
Clay (kaolinite, illite, montmorillonite)	Al_2_O_3_–SiO_2_ layered silicates	Shrinkage, moisture sensitivity, variability	Calcination, moisture control	Bricks, tiles, refractories	[107,108,110]
Modified clay systems	Clay + additives (lime, nanoadditives)	Agglomeration, processing sensitivity	Mechanochemical activation, curing	Stabilized soils, advanced ceramics	[114,125,126]
Composite systems (ash + slag + clay)	Multi-component oxide systems	Complex optimization	Pressing (10–25 MPa), sintering	Structural ceramics, lightweight materials	[51,52,61]

**Table 11:** Comparative environmental advantages of conventional and waste-based ceramic materials [146].

System	Environmental Impact	Advantages
Conventional ceramics	High CO_2_ and energy consumption	High mechanical stability
Waste-based ceramics	Reduced landfill and raw material use	Improved sustainability and circular economy

## Data Availability

No new data were created or analyzed in this study. Data sharing is not applicable to this article.
